# Supplementary material for: Causal Relationship between Aging and Anorexia Nervosa: A White-Matter-Microstructure-Mediated Mendelian Randomization Analysis
Source: Biomedicines. 2024 Aug 16;12(8):1874. doi: 10.3390/biomedicines12081874 (PMC11351342; doi:10.3390/biomedicines12081874)
Supplement: Supplementary file 1 [file biomedicines-12-01874-s001.zip › Supplementary Figure/Figure S4/Singe SNP plot of MR analysis for TL to ubm-b-2119.pdf]

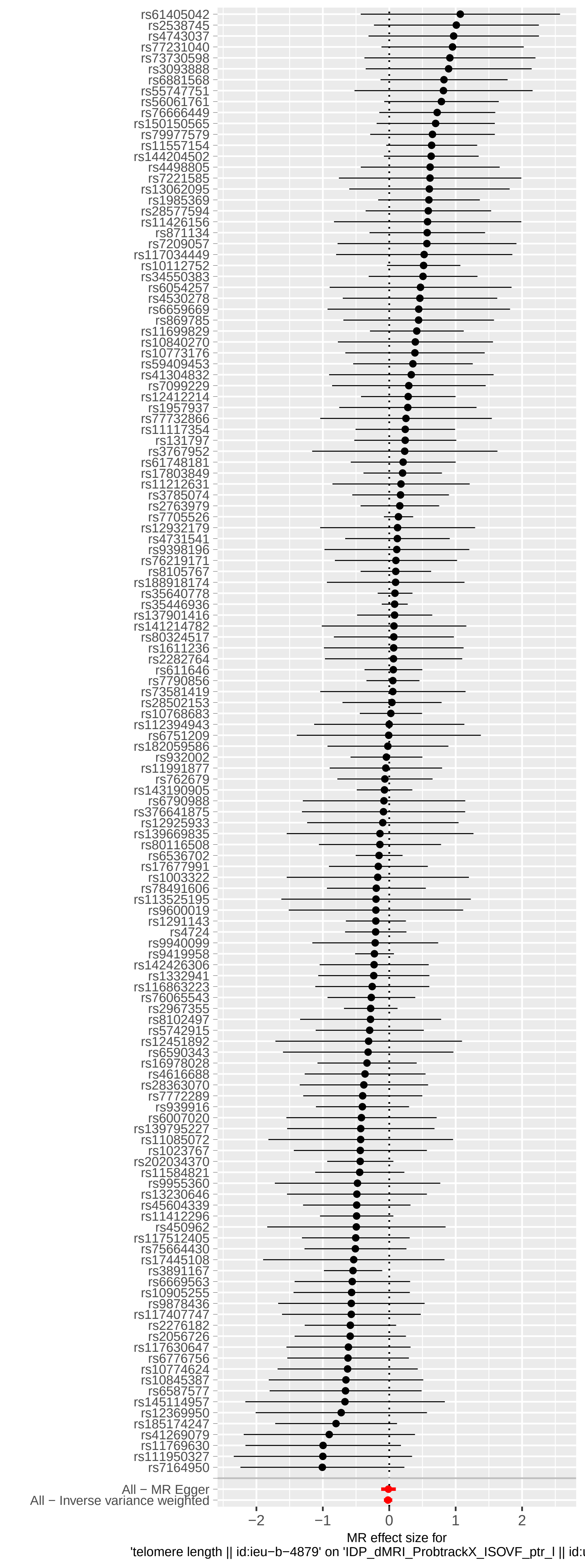

rs61405042
rs2538745
rs4743037
rs77231040
rs73730598
rs3093888
rs6881568
rs55747751
rs56061761
rs76666449
rs150150565
rs79977579
rs11557154
rs144204502
rs4498805
rs7221585
rs13062095
rs1985369
rs28577594
rs11426156
rs871134
rs7209057
rs117034449
rs10112752
rs34550383
rs6054257
rs4530278
rs6659669
rs869785
rs11699829
rs10840270
rs10773176
rs59409453
rs41304832
rs7099229
rs12412214
rs1957937
rs77732866
rs11117354
rs131797
rs3767952
rs61748181
rs17803849
rs11212631
rs3785074
rs2763979
rs7705526
rs12932179
rs4731541
rs9398196
rs76219171
rs8105767
rs188918174
rs35640778
rs35446936
rs137901416
rs141214782
rs80324517
rs1611236
rs2282764
rs611646
rs7790856
rs73581419
rs28502153
rs10768683
rs112394943
rs6751209
rs182059586
rs932002
rs11991877
rs762679
rs143190905
rs6790988
rs376641875
rs12925933
rs139669835
rs80116508
rs6536702
rs17677991
rs1003322
rs78491606
rs113525195
rs9600019
rs1291143
rs4724
rs9940099
rs9419958
rs142426306
rs1332941
rs116863223
rs76065543
rs2967355
rs8102497
rs5742915
rs12451892
rs6590343
rs16978028
rs4616688
rs28363070
rs7772289
rs939916
rs6007020
rs139795227
rs11085072
rs1023767
rs202034370
rs11584821
rs9955360
rs13230646
rs45604339
rs11412296
rs450962
rs117512405
rs75664430
rs17445108
rs3891167
rs6669563
rs10905255
rs9878436
rs117407747
rs2276182
rs2056726
rs117630647
rs6776756
rs10774624
rs10845387
rs6587577
rs145114957
rs12369950
rs185174247
rs41269079
rs11769630
rs111950327
rs7164950
All − MR Egger
All − Inverse variance weighted
−2
−1
0
1
2
MR effect size for
'telomere length || id:ieu−b−4879' on 'IDP_dMRI_ProbtrackX_ISOVF_ptr_l || id:i
